# Julie Zikherman receives the ASCI/Marian W. Ropes, MD, Award

**DOI:** 10.1172/JCI209204

**Published:** 2026-07-01

The American Society for Clinical Investigation (ASCI) honors Julie Zikherman, MD (Figure 1), with the ASCI/Marian W. Ropes, MD, Award. Dr. Zikherman is recognized for her outstanding research revealing the mechanisms of B cell tolerance that restrain autoimmunity and for her dedication to mentorship. She is an Associate Professor in the Department of Medicine at UCSF and serves as Associate Chief for Basic Research in the Division of Rheumatology. Among other leadership positions, she serves on the UCSF Physician-Scientist Scholar Program Steering Committee and the Physician-Scientist Career Development Program Advisory Council. Dr. Zikherman was elected to the ASCI in 2021. ASCI President-Elect Dr. Julie A. Bastarache, Associate Professor in the Department of Medicine, Allergy, Pulmonary and Critical Care Medicine at Vanderbilt University Medical Center, interviewed Dr. Zikherman at the Association of American Physicians/ASCI/American Physician Scientists Association Joint Meeting in Chicago in April 2026.

Julie A. Bastarache: Could you start out by telling us a little bit about yourself, your training path, and how you came to work on B cells and the other subjects you’re studying?

Julie Zikherman: It’s a pleasure. Well, my path was nonlinear. At the beginning, I didn’t know where I’d end up. I did my medical school training at Cornell. The medical school is in Manhattan, and I joined the tri-institutional MSTP [Medical Science Training Program] there. For my research, I became really interested in developmental biology and joined a lab that studied chick embryo development, [which had], at the time, minimal clinical relevance. I just followed what I thought was interesting, and a lot of developmental patterning seemed interesting. However, as time went on, my focus in the lab drifted, and I didn’t finish my PhD. I went back to clinical medicine [and] became really interested in rheumatology because of how it touches on every field in internal medicine. In other words, you don’t really give anything up; you have to think about every other disease that touches every other organ. And that’s where my real interest in the immune system arose.

I did my residency at Brigham and Women’s in Boston and started a fellowship there, then continued at UCSF. I had followed my husband-to-be across the country. And there I was really fortunate. There was an opportunity to join the lab of Dr. Art Weiss, who is a pioneer in T cell receptor signaling — so a completely different field. I’d been in a lab before, but all the techniques, all the approaches, the entire discipline was totally different. I basically had the chance to learn molecular immunology, flow cytometry, all the armamentarium of the modern immunologist as a fellow, which is a unique opportunity that we have in medical training in the US — that you can try a bunch of different things for a while until you find where your passion lies. And it turned out that I loved rheumatology and I loved immunology, and I had the opportunity to train deeply. There I got really interested in tolerance as the natural correlate of what we see in the clinic, but very basic mechanisms of tolerance. These are the things that are disrupted and perturbed when autoimmune diseases develop. And that’s true for any autoimmune disease, the ones that we treat in rheumatology, the autoimmune diseases that are mostly handled by other disciplines, and the ones that we cause, like checkpoint-induced irAEs [immune-related adverse events].

I started out with a focus on T cell development, central tolerance, and peripheral tolerance, and those are still interests. In fact, some of the features of T cell development in the thymus reminded me a lot of the things I’d loved about developmental biology — figuring out where blocks occurred during development. Those seemed analogous and familiar, and I enjoy them still. What kind of fate choices do T cells make as they’re developing so that you end up with a functional, tolerant repertoire, and how do signal-dependent checkpoints operate there? But during my time as a postdoc, two sequential projects, which I really enjoyed, each got scooped by a very similar publication that came out shortly before we were ready to publish. And I remember my mentor, who’s incredibly supportive, saying, “This won’t be the last time.” But what I gradually learned is that science isn’t about getting someplace first. It’s about thinking deeply about a topic and figuring out the nuances and pivoting a little bit. And the second such scoop — actually both of them — led us to think also about B cells, not just T cells, because the topics we were studying had relevance for both cell types.

It’s these pivots that drove me into B cell biology, even though I trained in a T cell lab. And ever since then, the parallels of the two cell types, the independent constraints that T cells and B cells face during their development and in their function later are revealing of what the design constraints of these systems are. What do you need to do to optimize how a T cell works? How a B cell works? Those constraints are the same things that establish the mechanisms that govern how they also distinguish self from foreign, in other words, fundamental mechanisms of tolerance in the adaptive immune system. Those are the kinds of things I’ve been passionate about that I continue to study, and I consider myself very fortunate to have made all these accidental choices. But I have to say that none of that would have happened, and certainly my career wouldn’t have happened, if I didn’t end up in the lab of Art Weiss, who is an amazing scientist and an incredibly supportive mentor, both while I was a postdoc and afterward. I think was the final reason I’ve ended up where I am now.

JAB: It’s so fun to hear about people’s paths because I don’t think anyone has ever had a linear path. You talked a little bit about Dr. Weiss. What other specific factors do you think were key to your success?

JZ: My path was long. I think I spent six years technically as a postdoc in Art’s lab. And even when I was starting my independent lab, I was highly reluctant to leave, because it was so much fun to be there — such great colleagues, such a supportive environment. And part of that is that I had switched fields completely, so I did a PhD and postdoc in one in some sense. But part of what I feel really fortunate about is that I had the chance to try multiple things, and I had the time to test out different ideas, to pursue different projects. Becoming a bench researcher takes a lot of time. It’s an enormous amount of training, to learn technically how to do things, but more importantly how to guide a project to fruition, how to understand what is going to lead somewhere exciting and what isn’t. And that’s something you can’t often tell at the very beginning. You have to go a certain distance along each project to sense that.

Having the time to train for that long set me up to have a better instinct about those aspects of a project when I became a mentor and had to guide students and postdocs along that path. At the time I started, the EMR [electronic medical records] didn’t exist yet, so I was able to have a clinical role but balance how much time that took and how much time I had to spend in the lab. I think that [was] really important. I ended up in a lab with, again, a mentor who I admire greatly, and over the course of all those years, I was able to absorb, What does great science look like? How do you push your observations to the next level? What does rigor look like? What does deep mechanistic insight look like? So the training wasn’t just doing the science, but it was hearing about everyone else’s projects, giving advice, exchanging ideas. Definitely my time with Art is what set me up to become a scientist, and without that I’m sure it wouldn’t have happened.

JAB: Mentors are so important. What obstacles did you have to overcome in this journey?

JZ: There were a number of false starts I alluded to at the beginning. One of my non-PhD projects when I was supposed to be finishing my MSTP focused on feather bud patterning in chick embryos, which I still think is fascinating. And you can find relevance in all kinds of ways, but I did feel like I’d reached a point where I couldn’t push that mechanistically any further. Part of it is that our lab at the time was studying chick embryos as a model system; it’s not a genetic model system. It’s amazingly powerful and was a workhorse organism for developmental biology, because you can create these little windows in fertilized eggs and you can watch the chick embryo develop under a not-powerful microscope. You can use retroviruses to misexpress genes, but you can’t knock anything out. It was the system where morphogen function was discovered, because you can put beads that secrete growth factors and then see what happens, like, “Now this chick has an extra digit, and this is what caused it.” It’s amazing, but I felt constrained.

The fact that our educational system allows us to try things, pivot, visit science again, reenter scientific training at multiple points — I think that’s unique in the US. The course of scientific training in other countries is a lot more streamed at earlier time points. For me it was important to try things until I found the right fit. Knowing when I wasn’t on the right path and being able and encouraged to pause and reassess was really important. I don’t know if you’d call that an obstacle; I think it would have been if I’d kept going. During my immunology training as a rheumatology fellow — I alluded to this earlier — I had multiple sequential projects. I think they built to a coherent focus, which is on understanding the mechanisms that control how T cells and B cells distinguish self-antigens from pathogens and foreign antigens. But as I mentioned, two of those projects did get scooped in surprising ways because they were both singular and strange approaches where you wouldn’t imagine that someone else was doing the exact same thing.

But I think those challenges forced us to stop and rethink and consider pathways around. What else can this tool be used for? What other approaches can we take? Those in a way also led me to interesting pathways in science. I have to say, in most ways I feel fortunate rather than challenged in the course of my education and training. Mostly I’ve felt very supported. Sometimes when I see my trainees now and appreciate the challenges that they’re facing, I think they’re new and different from what I had to overcome. The burden of clinical documentation is substantially larger. The forces that keep people out of the lab are more powerful. The pressures to do more and more high-throughput clinical medicine are more powerful. So they face a landscape where there are new challenges when you’re trying to navigate time for research, time for clinical work.

JAB: It is interesting, though, how sometimes, probably most of the time, a challenge ends up leading to even greater success. Where do you see your work going in the future?

JZ: Despite the fact that I’m a physician-scientist, our research has focused on extremely basic mechanisms of tolerance. Luckily, the immune system is relevant for maybe most of clinical medicine, extending far beyond autoimmunity per se. There is just a wealth of immune-mediated diseases, again, in other specialties, but this impacts how we think about how vaccines work, infectious diseases, cancer immunotherapy, transplant tolerance. It’s really this vast, interconnected network of human physiology and pathophysiology where tuning T cells and B cells is relevant. The sense in which being a physician-scientist has been important in immunology for me is seeing those connections. And one of the aspects of our work that I hope we can push forward is translating a little bit more some of the insights we have about how B cells make their fate decisions to inform how we can recruit them for protective immune responses, to target cancer, to reestablish tolerance in B cell–mediated autoimmune diseases. We’re still super-interested in fundamental signaling and transcriptional pathways that do this work. But I think it’s a really exciting time in medicine and biotech, because we have the tools to manipulate these pathways precisely, and there are no longer the same barriers we faced before, where you have to use a ligand for a cell surface receptor to do it.

We have degraders. There are no undruggable targets anymore. [There are] fundamental pathways at your disposal to manipulate. In my conversations with friends and colleagues who are pursuing drug development, what I’m always told is the constraints on even large biotech companies are not how to drug a target, but to figure out the right targets. As physician-scientists, we’re uniquely positioned to understand the diseases, to figure out the pathways that make the most sense, and hopefully we can collaborate across the spectrum to figure out how we can even repurpose existing therapies.

JAB: Your work is a beautiful example of how fundamental basic biology discoveries underlie the clinical impact that potential therapies could have. One last question. What advice would you have for people who want to become physician-scientists?

JZ: This is informed a lot by my time as a mentor and trainees in the lab: I would say that you have to have grit and perseverance, that the path can take a while, and there are a lot of areas where you can feel discouraged, but you have to keep going. Trainees who are fellows entering a lab who have several years before they’re able to transition to independence, for example, not only will face challenges in their science, but also even getting training grants. The key is to gradually build up a thick skin, keep resubmitting your proposals, hang in there. Beauty is in the eye of the beholder, and sometimes you have to submit and submit and submit until someone can appreciate the potential of the work you’re doing. Sometimes you can get lucky early on, but even if you do at the very beginning, you’ll still hit this phase where you have to keep trying sooner or later.

I think I got fairly lucky with early-training grant funding, probably because I was in the lab of a world-renowned T cell receptor biologist, and I benefited from that. But when I set out on my own and was trying to get my first R01, I also got a triaged grant, read the summary statement, [went to] hide in a corner. But even at that point, I got great advice: Just keep going. Whatever obstacles you meet, there’s usually a way around. You just have to pause and think about it from a different standpoint. It’s an amazing opportunity to go to work and have the chance to explore how nature works. And I’m mindful of that, or I try to be. I think we all have our discouraged days. To just step back and see that you have the chance to do something unique and powerful and take rejection in stride — whether it’s manuscript rejection, grant rejection — and keep going.

JAB: It really is an amazing career. Thank you so much, and congratulations on receiving this award.

JZ: Thank you. I’m honored to receive it.

*The interview has been edited for length and clarity.*

## Figures and Tables

**Figure 1:**
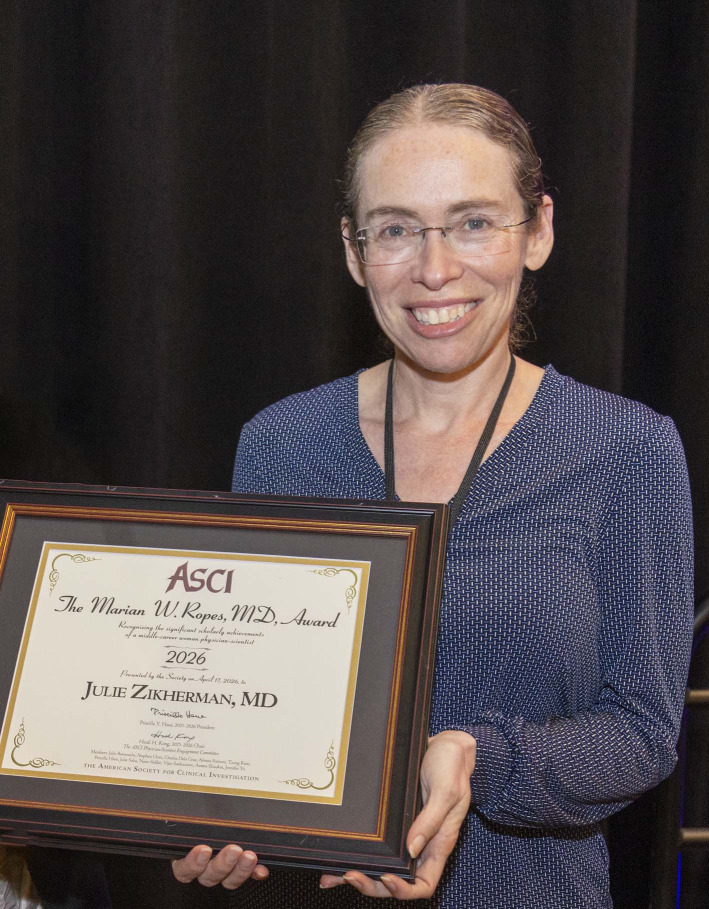
Julie Zikherman is the recipient of the 2026 Marian W. Ropes, MD, Award.

## Version 1 07/01/2026

Electronic publication

